# The world’s largest cities under climate change and their adaptive capacity to rising heat

**DOI:** 10.1038/s41598-025-19954-z

**Published:** 2025-09-23

**Authors:** John Friesen, Hannes Taubenböck

**Affiliations:** 1 Department of Global Urbanization and Remote Sensing, University of Wuerzburg, John-Skilton-Str. 4a, 97074 Würzburg, Germany; 2 German Remote Sensing Data Center (DFD), German Aerospace Center (DLR), Oberpfaffenhofen, Germany

**Keywords:** Climate change, Urbanization, Climate niche, Exposure, Projections, Environmental impact, Climate-change impacts, Sustainability

## Abstract

We quantify future urban heat exposure and adaptation capacity for the 1563 largest global cities, for the first time globally integrating climate projections, urban morphology, and economic capacity. We use high-resolution mean annual temperature (MAT) projections under SSP1-2.6, SSP3-7.0, and SSP5-8.5. These are combined with Local Climate Zone (LCZ) profiles and downscaled socioeconomic data, evaluated consistently within morphological city boundaries. With this framework, we identify cities projected to exceed a 29 °C MAT threshold by 2071–2100. The number of threshold-exceeding cities is projected to rise from 17 (2011–2040) to 217 (2071–2100), exposing up to 320 million residents. Cities with compact built-up forms show higher exposure, while responsiveness to eight expert-curated adaptation measures (e.g., reflective materials, greening, water bodies) and GDP distributions reveal large regional disparities in adaptive capacity. European cities face the steepest relative warming (median + 4 °C under SSP5-8.5), while African and South American cities, despite smaller increases (+2.7 to 3.2  °C), confront higher baseline heat. Our framework demonstrates how morphology- and economy-informed adaptation planning can spatially target measures to safeguard urban habitability in a warming world.

## Introduction

Since 2008, the majority of the world’s population is living in cities, which now accommodate over 4 billion people and continue to expand unevenly under diverse socio-economic and environmental pressures^1,2^. Urban areas exhibit distinct warming dynamics, driven by factors such as heterogeneous land-use patterns and high levels of imperviousness. These dynamics amplify heat stress and put a strain on different urban infrastructures (water supply, public health systems, etc.)^3^. Beyond acute extremes, urban residents, services, and economies experience heat primarily as a persistent background burden; mean annual temperature (MAT) is therefore a meaningful indicator of urban heat stress because it summarizes chronic exposure that drives cooling demand, baseline health risks, and day-to-day labor productivity^4^. Rising background heat can erode productivity and livability and, where services are already strained, may also influence migration decisions^5^.

The climate-niche framework^4^, which characterizes, among other parameters, the range of MAT within which most people have historically lived, provides a concise lens for assessing when and where future warming will push cities beyond historically comfortable conditions. A MAT threshold of 29 °C is frequently adopted as an upper bound for global habitability assessments, though exploring alternative thresholds (e.g., 27 °C) can reveal additional vulnerabilities. We include 27 °C as a conservative comparison to test sensitivity and to reflect heterogeneity in human thermal tolerance and adaptation: recent analyses indicate that potentially lethal heat exposure (> 40 °C) rises sharply once MAT exceeds  27 °C and becomes widespread by 29 °C^5^. This linkage between background climate and the frequency of dangerous extremes provides physiological and empirical rationale for including 27 °C as a sensitivity benchmark, even as our emphasis remains on sensitivity rather than a hard habitability limit.

### Literature review

Xu et al.^4^ were among the first to formalise the human climate niche using historical data, alongside others (e.g.^6^). They fitted a double-Gaussian model to the global population-mean annual temperature (MAT) and precipitation (MAP) distribution, thereby establishing a quantitative baseline for future comparison^4^. Building on this, Lenton et al.^5^ applied climate projections and SSP (shared socioeconomic pathways) population scenarios to estimate that under SSP2-4.5 roughly 40% ($\approx$3.7 billion) of people would lie outside the historic niche by 2100 and that this share rises above 50% under high-emission pathways while limiting warming to 1.5 °C could reduce niche departures to $\approx$28% ($\approx$2.7 billion)^5^. These population-based studies clarify where people may move outside historically familiar climates, but they abstract from intra-urban morphological heterogeneity that modulates local heat exposure.

Parallel studies have integrated urban growth and heat extremes: Taubenböck et al.^7^ delineated 1567 morphological urban areas using the World Settlement Footprint, projected settlement expansion to 2045, and identified hotspots for extreme rainfall and temperature exposure. Tuholske et al.^8^ harmonised high-resolution temperature and humidity data for 13,115 urban settlements (1983–2016) and found a 199% increase in person-days of wet bulb globe temperature higher than 30 °C, driven by population growth (two-thirds) and warming (one–third). The 2024 World Cities Report^3^ utilised the Urban Cities Database with SSP scenarios to analyse transitions in climate zones and vulnerabilities to multiple climate-related exposures until 2045.

Taken together, prior work either projects global populations and the climate niche through 2100 (e.g.^4,5^) or assesses climate exposures for many cities over shorter horizons (e.g.^3,7,8^), but typically with (i) inconsistent or administrative boundary definitions rather than consistent morphological extents, (ii) limited treatment of intra-urban land-cover heterogeneity, and (iii) no explicit assessment of adaptive capacity.

### Research contribution

We present the first global-scale integration of climate-niche assessment with consistent morphological urban boundaries, LCZ-based intra-urban morphology, and a socioeconomic proxy for adaptive capacity. In contrast, whereas Xu et al.^4^ focus on population-climate distributions and Lenton et al.^5^ on niche departures by population, we map these assessments onto consistent morphological urban extents and their LCZ structures, linking exposure to LCZ-specific adaptation options and to the capacity to implement them.

**Fig. 1:**
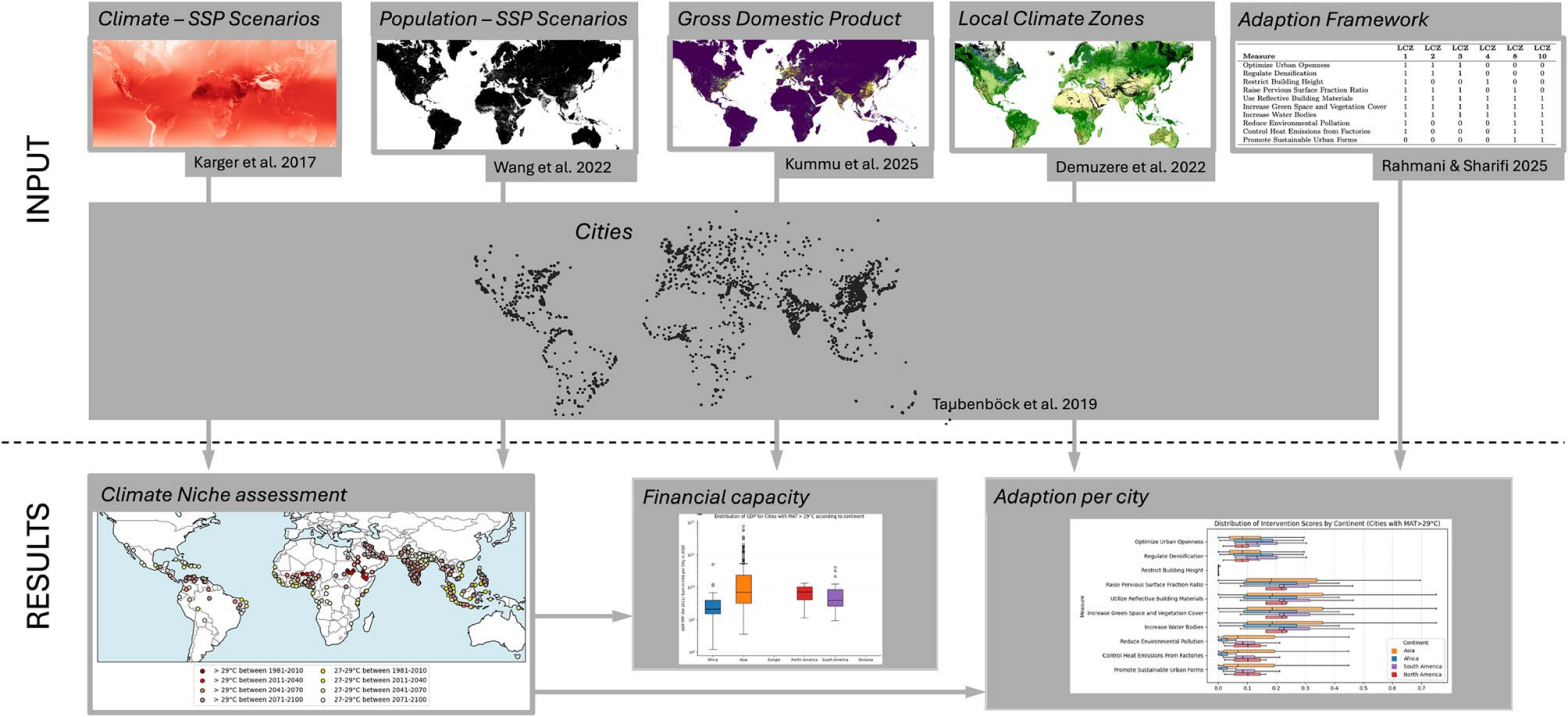
Framework of this study. Grids with 1 km resolution of climate^13^, population SSP scenarios^10^, GDP for the year 2020^12^ and LCZ with 100 m resolution^9^ are clipped down to the morphological urban areas derived by Taubenböck et al.^1^. These citywide aggregated metrics are then analyzed and the share of LCZ is combined with the adaptation matrix in Table 1. Detailed information is available in the Methods section. The maps in the upper and middle row of the figure were created by visualizing the input data^1,9,10,12,13^ in QGis (Version 3.36). The lower row shows exemplary results presented later in the paper (Figs. 3B, 6A,C).

Conceptually, we combine downscaled mean annual temperature projections with SSP population scenarios within 1563 morphological urban areas^1^, identify cities projected to exceed 29 °C MAT by 2071–2100 (with 27 °C as a sensitivity), quantify exposed populations, and characterise the LCZ share profiles of exceeders across regions^9,10^. We then translate those LCZ profiles into a typology of recommended measures using an expert-based adaptation framework^11^. Finally, we overlay a gridded Gross Domestic Product (GDP) proxy^12^ to approximate adaptive capacity, enabling an equity-sensitive view that connects urban form not only to *exposure* but also to the *capacity to act*. The conceptual framework of this study is shown in Fig. 1.

Our outputs are: (i) a global, scenario-specific screening of cities by MAT threshold exceedance and exposed population; (ii) regionally summarised LCZ-specific adaptation typologies with morphology-led prioritization cues; and (iii) a combined morphology-capacity lens to support tailoring adaptation strategies to where they are most needed.

### Research questions

By using the presented methodology, we are able to answer the following reasearch questions:

1. **How will mean annual temperatures evolve** in 1563 major cities (all cities accross the globe with more than 300 000 inhabitants) under SSP1-2.6, SSP3-7.0, and SSP5-8.5, and how do these trends vary across world regions?
2. **How will population change and intra-urban morphology (LCZ)** jointly shape exposure to MAT threshold exceedance (29 °C; 27 °C)? Which cities and regions concentrate the largest exposed populations, and which LCZ share profiles are over- or under-represented among exceeders?
3. **Which LCZ-specific adaptation measures** follow from the assessed LCZ profiles associated with threshold exceedance, and how should these measures be prioritized by morphology given projected warming and demographic shifts?

## Results

**Fig. 2:**
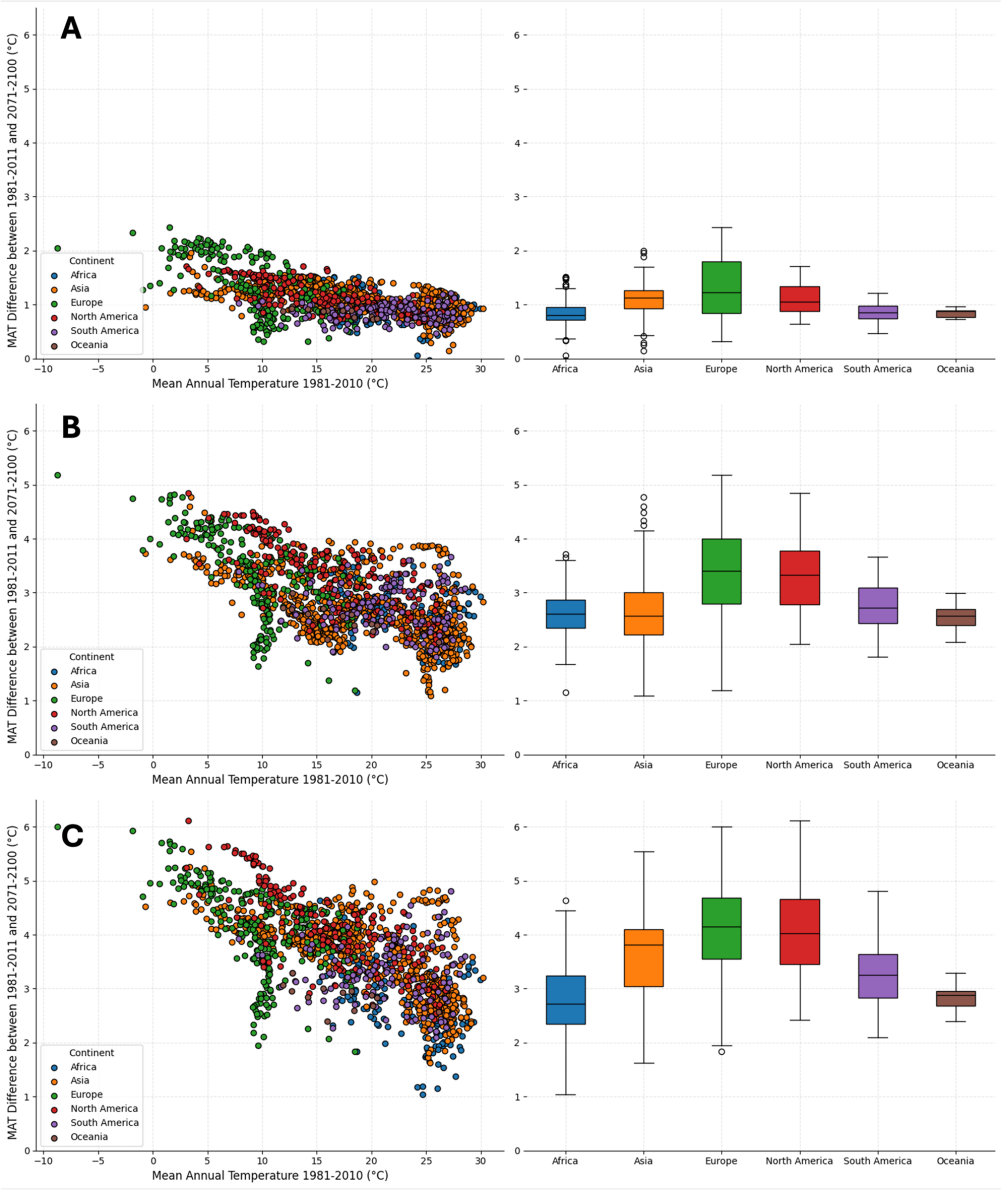
Differences in mean annual temperature in comparison to reference period. (**A**) Distribution of changes in mean annual temperature in comparison to the reference time span 1981–2010 per continent and box plots per continent for SSP1-2.6, (**B**) SSP3-7.0 and (**C**) SSP5-8.5. The figures were created using the code that can be found in the repository, as stated in the code availability statement.

Our findings show significant regional variations in temperature increases. The cities in Europe exhibit on median the strongest warming trends in all scenarios considered. According to SSP5-8.5 (Fig. 2C), more than 50% of European cities are projected to experience an increase of the MAT of more than 4.1 °C between 2010 and 2100. North American cities follow closely, with a median increase of 4.0 °C. Asian cities exhibit a slightly lower temperature rise, with a projected average increase of 3.8 °C. In contrast, cities in South America and Africa are projected to experience comparatively lower temperature increases, with averages of 3.2 °C and 2.7 °C, respectively. Even if these latter predicted temperature increases are lower, it must be borne in mind that the average initial temperatures e.g. in Africa are already on a high level, often already close to the upper end of the climate niche. Similar diagrams for the other two scenarios (SSP1-2.6 and SSP3-7.0) are shown in Fig. 2A,B.

**Fig. 3:**
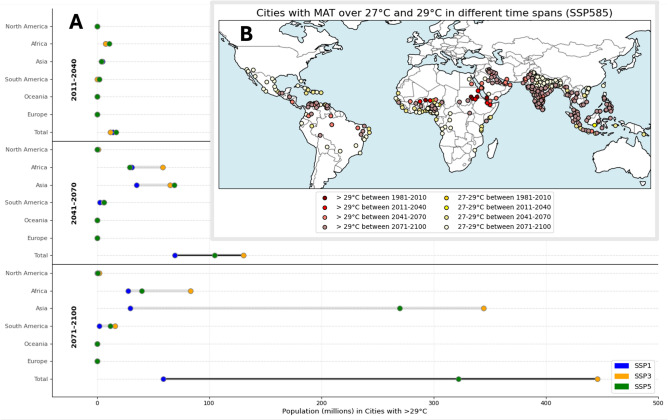
Population numbers and spatial distribution of cities exceeding a MAT of 29 degree Celsius. (**A**) Projected populations in cities with MAT >29 degree Celsius for different continents based on various climate projections. (**B**) Geographical distribution of cities with MATs between 27 and 29 and over 29 degrees according to SSP5-8.5. The figure and map were created using the code (Python 3.9 and the *cartopy* package) that can be found in the repository, as stated in the code availability statement.

To better understand the implications for urban populations, we apply the concept of the human climate niche, as described in previous studies^4^. The niche, mainly defined by areas with a MAT below 29 °C^5^, is critical for human habitability and well-being. According to SSP5-8.5, currently, 17 cities - ten African, six Asian and one South American - fall outside the habitable temperature range for the period 2011–2040 (Fig. 3B).

**Fig. 4:**
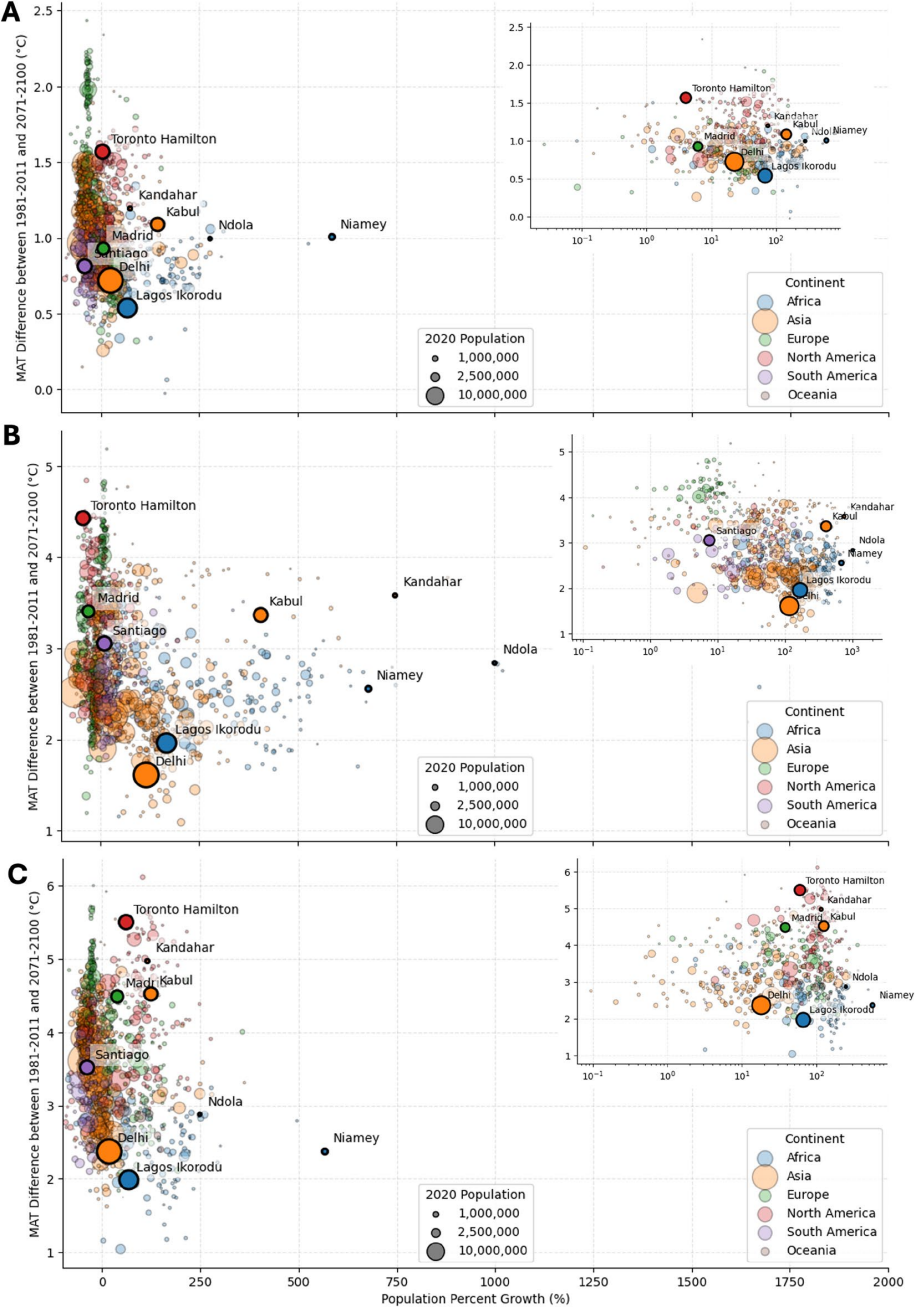
Differences in mean annual temperature and relation to urban growth. (**A**) Distribution of cities based on the difference in MAT and the population growth in linear and in the small plot on a logarithmic scale for SSP1-2.6, (**B**) SSP3-7.0 and (**C**) SSP5-8.5. The figures were created using the code that can be found in the repository, as stated in the code availability statement.

However, by 2041–2070, this number is projected to increase to 57 cities, with a combined population of 105 million (SSP5-8.5). For the final time period, 2071–2100, the situation becomes even more concerning, with 217 cities projected to fall outside the human climate niche. The total projected population for cities above 29 °C is 320 million people (SSP5-8.5). The majority of these estimated populations will reside in Asian (270 million), African (40 million) and South American (12 million) cities (Fig. 3A). Even in the most favorable scenario (SSP1-2.6), still more than 59 million (0.8% of projected world population in SSP1-2.6) people worldwide are projected to live in major cities with a MAT of more than 29 degrees, and more than 446 million people (3.5% of projected world population in SSP3-7.0) in the worst scenario (SSP3-7.0) (Fig. 3A). It is also striking that even in the best scenario (SSP1-2.6), almost 70 million people will live in cities with a MAT of more than 29 degrees by the middle of the century (2041–2070).

To analyze the sensitivity of our results, we perform the same analysis for slightly lower MATs. If we set a threshold of 27 °C—below the critical climate niche yet still concerning—by 2041–2070, approximately 221 cities are projected to experience MATs between 27 and 29 °C, impacting around 385 million people in the SSP585 scenario. By 2071–2100, this decreases slightly to 197 cities, with a projected population of 307 million. For comparison, projections range from a lower boundary of 195 cities affecting 362.5 million (SSP1-2.6) to an upper boundary of 205 cities with 639 million people (SSP3-7.0) by century’s end, highlighting the varying yet significant impacts across scenarios.

When the threshold is reduced to 27 °C, the total population exposed to these temperatures is projected to rise significantly, reaching 629 million people in the SSP5-8.5 scenario. Across all scenarios, this equates to a range of 357–1085 million people, or approximately 5.1% of the estimated global population in SSP1-2.6, 8.5% in SSP5-8.5, and as high as 8.5% in SSP3-7.0.

In Fig. 4A–C, the differences in MAT and population growth for all cities are shown for each of the three analysed scenarios. Under SSP5-8.5, projected urban population trajectories exhibit pronounced regional contrasts (Fig. 4C). African cities are the fastest-growing, though growth rates are small relative to SSP3-7.0 (Fig. 4B); Niamey leads with a 586% increase by 2100. North American metropolitan areas, which are expected to grow by only 0–10% under SSP3-7.0, are expected to show an expansion of roughly 10–100% in SSP5-8.5, reflecting the sensitivity of demographic outcomes to socioeconomic assumptions (Fig. 4C). South American and Oceanian cities cluster at the lower end of the spectrum, with several South American and Oceanian cities even exhibiting slight declines.

In South Asia, Delhi’s population rises by 10–20% under SSP5-8.5, a stark moderation compared to the 400–750% booms projected for Kabul and Kandahar under SSP3-7.0 (Fig. 4B). Nevertheless, Kabul and Kandahar still double their populations ($\approx$100%) in SSP5-8.5, compounding one of the largest MAT increases in our analysis (4–5 °C; Fig. 4C). These paired demographic and thermal pressures could intensify if alternative pathways (like SSP3-7.0 - Fig. 4B) unfold. Delhi’s more modest growth belies acute vulnerability: baseline MATs already exceed 25 °C, and warming of > 2 °C under SSP5-8.5 will further stress urban livability.

These projections underscore both the heterogeneous nature of future urban expansion and the uncertainties embedded in long-term scenarios. The differences between SSP1-2.6 (Fig. 2A), SSP3-7.0 (Fig. 2B) and SSP5-8.5 (Fig. 2C) highlight that adaptation strategies must remain flexible to accommodate a wide range of possible urban futures. They also show the heterogeneous nature of urban growth and underscore the varying challenges cities in different regions will face.

**Fig. 5:**
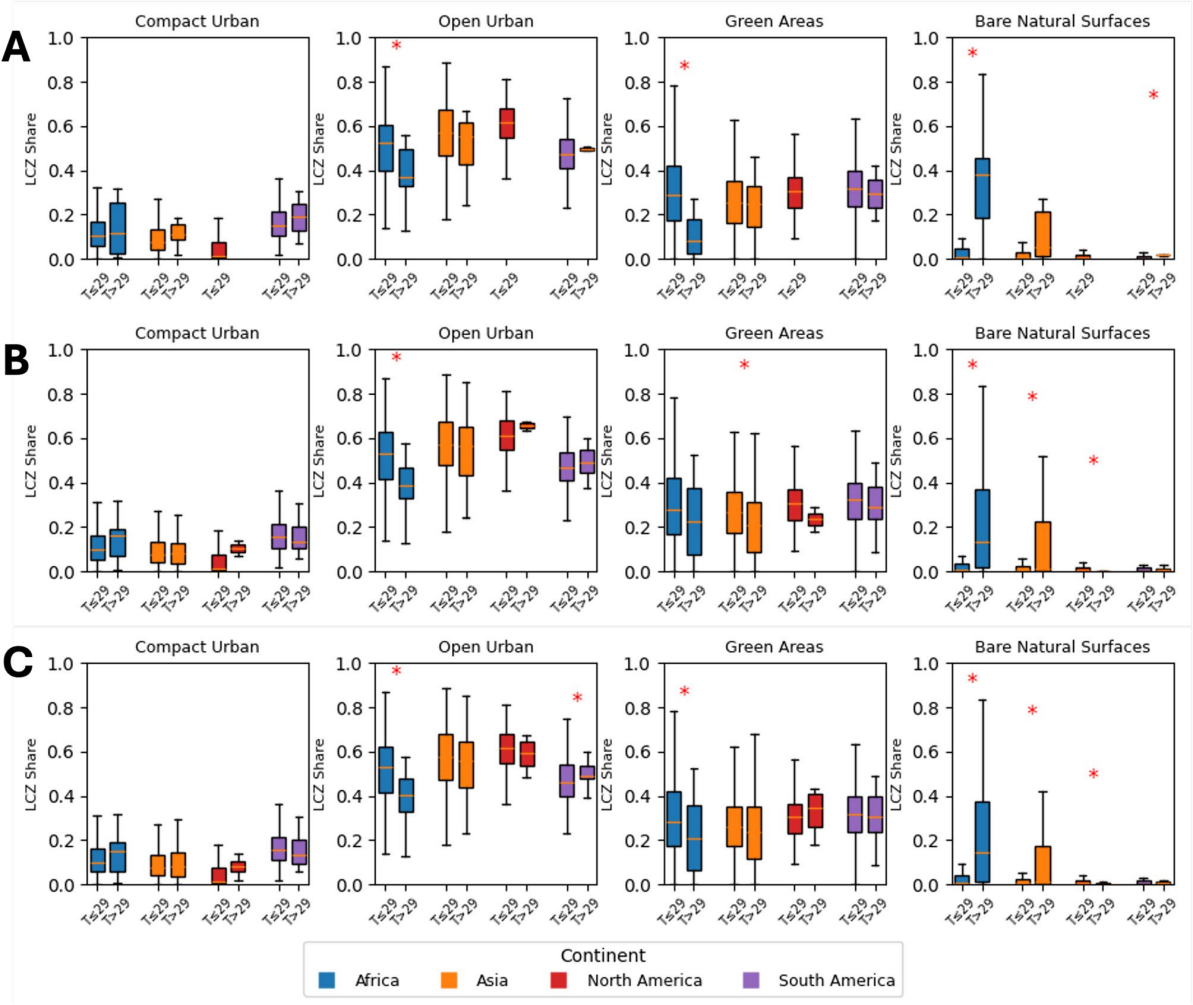
Boxplots showing the differences between the share of LCZs in the investigated cities. The cities are divided in two groups for each continent, one with higher and one with lower MATs than 29 degree Celsius. The order of continents is the same for all subfigures. The figures in A are created based on the SSP1-2.6 scenario, figures in B based on the SSP3-7.0 scenario and figures in C based on the SSP5-8.5 scenario. The figures were created using the code that can be found in the repository, as stated in the code availability statement.

Our analysis of local climate zones (LCZs) uncovers distinct urban morphological patterns in cities projected to exceed a 29 °C mean annual temperature (MAT) threshold (Fig. 5). While the share of compact urban areas is comparatively low in every city across both groups in all global regions, African cities below the 29 °C threshold display significantly larger open urban spaces in all considered scenarios. Moreover, in Africa, Asia, and North America, cities with MAT > 29 °C exhibit a notably higher proportion of bare natural surfaces (in SSP3-7.0 and SSP5-8.5), potentially intensifying thermal stress. In Africa, these hotter cities also possess a significantly lower share of green spaces in SSP1-2.6 and SSP5-8.5. Collectively, these findings suggest that urban forms in cities surpassing the human climate niche are characterized by specific features that may exacerbate heat exposure, underscoring critical avenues for targeted adaptation strategies.

**Fig. 6:**
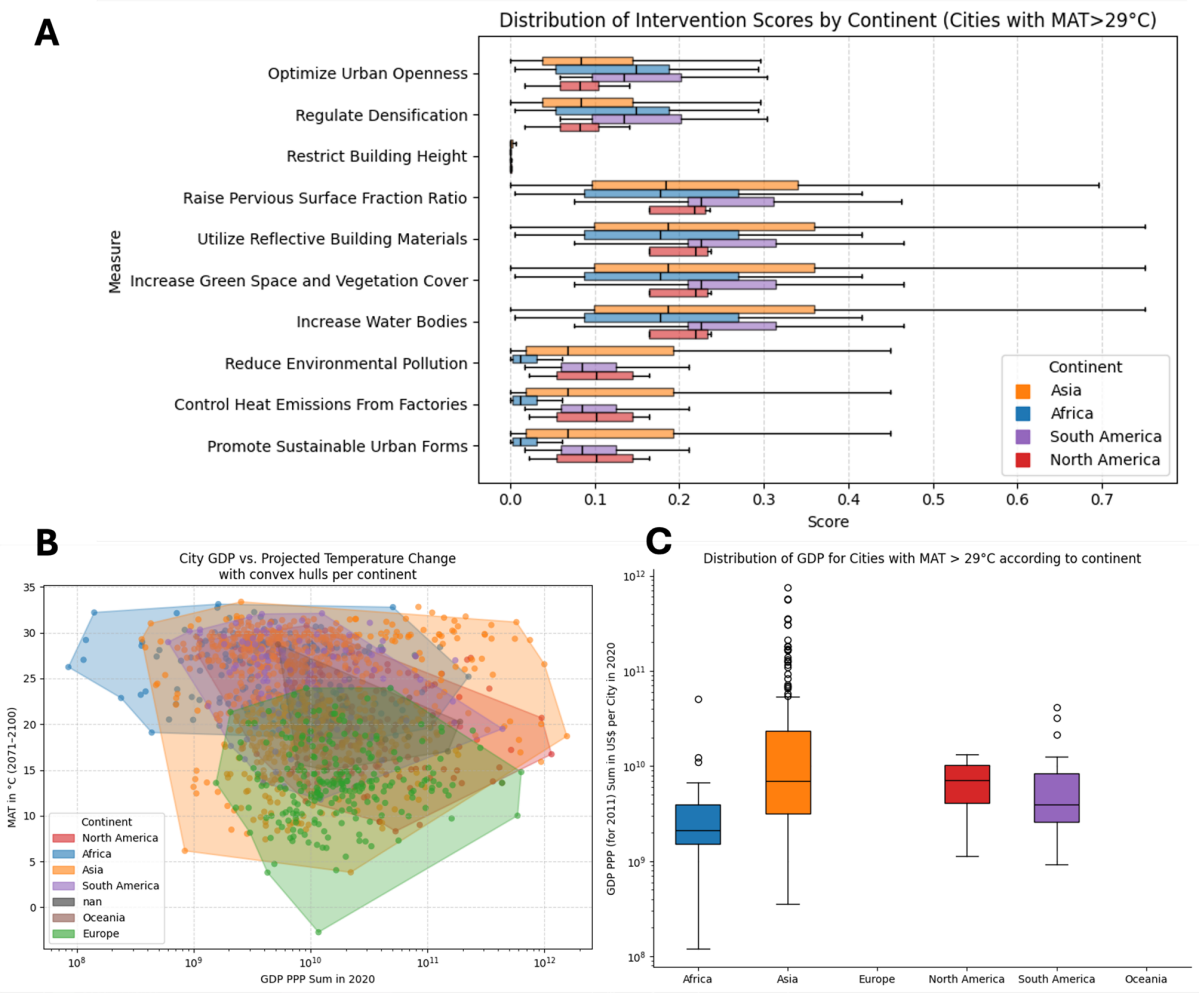
Climate adaption measures and financial capacity. (**A**) Distribution of scores of different adaption measures for cities in different continents exceeding MAT of 29 °C. The scores reflect the LCZ distributions of the respective cities. (**B**) Gross Domestic Product based on purchasing power parity (GDP PPP) sum for each morphological area clustered by continent in relation to the MAT (2071–2100). (**C**) Boxplots describing the GDP PPP sum for cities per continent for all cities with MAT higher 29 °C. The figure is based on SSP5-8.5. The figures were created using the code that can be found in the repository, as stated in the code availability statement.

As a final step, we present, based on a literature review by Rahmani and Sharifi^11^, various adaptation strategies for cities with an expected MAT higher than 29 °C. The authors analyzed which adaptation strategies best fit specific LCZ classes. This approach was applied to all cities, with the measures assigned according to the respective LCZ’s share of the total city area. The greater the proportion of a particular LCZ in a given city, the greater the score assigned to the corresponding adaptation measures (Fig. 6A). Considering the distribution of LCZs across different cities, we found that increasing the pervious-surface fraction, using reflective building materials, expanding greenspaces and vegetation cover, and enhancing water bodies scored highest in all continents. However, there is substantial variation among cities: for instance, the suggested pervious-surface-fraction increase is zero in Chertala (India) but reaches 0.75 in Kuwait City (Kuwait). Satellite imagery shows Cherthala’s extensive greenery driven by its tropical monsoon climate and abundant rainfall, whereas Kuwait City’s near-absence of vegetation reflects its hyper-arid desert climate, minimal precipitation and reliance on expensive desalinated water for any irrigated plantings.

Because high-rise areas (LCZ 1 and LCZ 4) occupy only a small share of most cities, restricting building height is not a priority intervention. Likewise, since LCZ 8 and 10 are scarce in African cities, measures to reduce environmental pollution or control industrial heat emissions score low in those contexts. In contrast, our methodology assigns high scores to these interventions in Muscat (Oman) due to its substantial LCZ 8 coverage. City-specific scores are detailed in the Supplementary Data.

To assess each city’s capacity to implement these changes, we related the projected MAT in 2100 to GDP^12^ per city (Fig. 6B), focusing on cities exceeding 29 °C MAT (Fig. 6C). Asian cities exhibit the greatest GDP variability. The MAT of European cities remain below the threshold of 29 °C MAT and therefore no city is plotted in Fig. 6C, although these cities show comparably high GDP values. By contrast, although African cities have the highest MAT values, they generally have the lowest GDP levels.

## Discussion

Our analysis indicates that rising urban temperatures will have profound implications for the future livability of cities and confirms previous studies on this topic^3,7^. Under SSP5-8.5, up to 217 of today’s largest cities (>300,000 inhabitants) could exceed the 29 °C MAT threshold by 2071-2100, affecting $\sim$320 million people. Cities sort into three recurring risk profiles: (1) already-hot megacities where exceedance intersects with very large exposed populations (e.g., Mumbai, Chennai); (2) temperate but dense legacy cities facing steep background warming despite slower growth; and (3) hot-and-growing cities with constrained adaptive capacity, where rapid urbanization and informality amplify exposure.

Across threshold-exceeding cities, intra-urban morphology systematically modulates exposure. From 217 cities, 61.3% have bare natural surfaces above their continent-specific median, 57.6% have green-area fractions below the median, and 58.1% have low-plant shares below the median. LCZ profiles of exceeders show higher shares of bare natural surfaces and lower vegetated fractions relative to non-exceeders (Fig. 2), consistent with greater heat absorption and reduced evapotranspirative cooling. In Africa, exceeders tend to have markedly lower green-area shares; in Asia, the reduction is concentrated in low-plant LCZs. Conversely, cities with larger pervious and vegetated fractions show damped exposure—pointing directly to morphology-leveraged adaptation opportunities.

In dense, cooler-climate cities (e.g. in Europe or North America), the median projected MAT increase exceeds 4.1 °C by 2100 under SSP5-8.5 (Fig. 2)—a large shift for infrastructures designed for milder conditions^14^. In many hot-and-growing cities, baseline MATs are already near or above threshold; rapid population increases magnify exposure—e.g., up to 600% growth in some cases (and 1500% under SSP3-7.0). Even where populations stabilise or decline (e.g., some North American and South American cities), high densities mean that a $\sim$ 4 °C rise in MAT will still exacerbate urban heat risks.

Delhi’s large informal-settlement share^15^ heightens vulnerability as background heat rises; Kabul’s potential expansion (up to 400%, scenario-dependent) combines rapid demographic change with increasing thermal stress. Madrid and Belgrade face steep warming atop dense, legacy-built fabrics. Toronto’s exposure grows with or without population increase due to high population numbers. Santiago’s dense informal areas face amplification under notable warming^16^. These examples reflect the three profiles mentioned above.

Linking LCZ diagnostics to our adaptation results (Fig. 6A), four measures consistently rank highest^11^: increase pervious-surface fractions; deploy reflective building materials in compact/dense LCZs; expand greenspaces/vegetation; and add or enlarge urban water bodies for evaporative cooling and co-benefits. Local feasibility varies: some Asian cities combine high MAT projections with limited GDP (Fig. 6B,C), constraining large-scale greening or retrofits. In wealthier metropolises (assuming political and societal will), reflective roofs and permeable pavements can scale rapidly. The contrast between Cherthala (high vegetative baseline, low pervious-surface score) and Kuwait City (desert-locked, high pervious-surface potential) illustrates how climate context, LCZ composition, and water resources shape feasible portfolios.

Thematically, the three profiles suggest different policy mixes: (1) for already-hot megacities, near-term heat action plans, reflective-surface mandates, and targeted greening in the hottest LCZs; (2) for temperate-but-warming dense cities, code updates for cool materials, retrofitting programs, and climate-fit species selection in parks; (3) for hot-and-growing, capacity-constrained cities, low-cost LCZ-targeted measures (cool coatings, shade trees, permeable lanes) and investments in basic services that also cool (water and sanitation). Further studies should also analyse which financing mechanisms best suit the different types of adaptation measures and risk profiles.

Together, these findings indicate that *who* is exposed (population trajectories), *where* exposure concentrates (LCZ patterns), and *what* is feasible (adaptive capacity) co-determine risk. Framing results by risk profiles avoids regional repetition and clarifies how morphology can either amplify or damp projected warming, while pointing to LCZ-informed, finance-aware pathways for implementation.

### Limitations

It is important to acknowledge that the concept of the human climate niche is inherently complex and contested^17^, even if it remains useful for showing global trends. While the $\hbox {MAT} =$ 29 °C threshold provides a benchmark^5^, urban habitability is influenced by multiple interacting factors—including humidity, access to resources, and socioeconomic conditions—so results should be interpreted as indicative rather than deterministic.

***Climate data*** Our study relies on downscaled climate projections (CHELSA, 1 km resolution), which are not designed to capture urban heat island (UHI) effects. As such, they may underestimate microclimatic variability where impervious cover and sparse vegetation amplify thermal stress. Local UHI spikes of 1–5 °C above surrounding areas^3^, when combined with humidity, can push MAT and wet-bulb thresholds beyond critical levels in low-greenness neighborhoods^18^. Although MAT >29 °C provides a broad niche indicator^4^, future urban analyses should incorporate wet-bulb metrics, humidity patterns, and high-resolution urban climate models or observations. Another limitation is that only a subset of CMIP6-SSP combinations is available at CHELSA’s native resolution; notably, SSP2-4.5 is missing. Inclusion of additional pathways would help test sensitivity to intermediate emissions scenarios.

***Demographic projections*** Population estimates are drawn from downscaled SSP products for consistency with climate inputs, but demographic projections vary in both totals and spatial allocation. Alternatives such as WorldPop use different urbanization algorithms that could shift local exposure estimates^19^. Moreover, even current counts—particularly for residents of informal settlements—are incomplete^15^. Future work could improve coverage by integrating high-resolution slum maps, household survey data, and sensitivity analyses across multiple population datasets.

***LCZ classification and land-use change*** Adaptation recommendations are tied to Local Climate Zone (LCZ) profiles, which capture the distinct morphological characteristics of urban neighborhoods. However, uncertainties in LCZ mapping stem from remote-sensing resolution and seasonal land-cover dynamics. More critically, LCZs are assumed static, although infill development, densification, or greening initiatives will change their distribution over time. Periodic updates and scenario testing are therefore needed to ensure that adaptation strategies remain aligned with evolving urban morphologies.

***Adaptation scoring*** The assignment of measures to LCZ classes follows a binary scheme, which oversimplifies suitability. A more nuanced, weighted approach would better reflect gradations in effectiveness across settings. Additionally, we did not account for existing city heat action plans, which may already address some of the recommended measures.

***Economic capacity*** Finally, we approximate adaptation capacity using total GDP per city. While useful as a first proxy, GDP does not capture institutional strength, governance quality, or socio-economic vulnerability. Incorporating per-capita indicators, governance metrics, and vulnerability indices could provide a more complete assessment of adaptive capacity.

## Conclusion

This study offers a comprehensive, city-scale evaluation of future urban thermal risks under three emissions scenarios (SSP1-2.6, SSP3-7.0, SSP5-8.5). We found that European cities are projected to experience the largest increases in mean annual temperature often exceeding 4 °C by 2100 under SSP5-8.5. While African and South American cities already operate at much higher baseline MATs, they show smaller temperature rises (2.7–3.2 °C). As a result, the number of municipalities exceeding the 29 °C “human climate niche” threshold grows from 17 in the near term (2011–2040) to 217 by century’s end, placing up to 320 million urban dwellers at risk. These live predominantly in Asia and Africa according to SSP5-8.5.

Our analysis of Local Climate Zones (LCZs) reveals that urban morphology strongly correlates with thermal exposure. Statistical tests confirm that cities projected to surpass the 29 °C threshold have significantly different LCZ compositions (*p* < 0.05) from cooler counterparts. By mapping these LCZ shares against an expert-curated adaptation-strategy matrix, we show that four measures (raising pervious-surface fractions, deploying reflective materials, expanding green cover, and enhancing urban water bodies) consistently score highest across continents.

Crucially, the feasibility of these interventions depends on local economic capacity. Gridded GDP (PPP) sums at the city level reveal wide disparities within and across continents. African cities with the highest MAT baseline show lower mean GDP values than cities of other continents. This points to the need for targeted, equity-focused policies, international cooperation, and capacity building to ensure that financially constrained cities which are often those facing the greatest heat burdens can implement effective adaptation measures.

Looking ahead, urban adaptation planning should be dynamic and data-driven. As city form, population distributions, and climate projections evolve, periodic updates to LCZ maps, gridded population data sets, and climatic thresholds will be essential. Integrating humidity and wet-bulb temperature data, conducting formal sensitivity analyses across multiple demographic and LCZ datasets, and extending our framework to smaller cities will further refine exposure estimates. Finally, embedding species-specific vegetation models into urban greening strategies can optimize cooling benefits under local climatic constraints. By explicitly linking high-resolution climate projections, urban morphology, and socioeconomic capacity, our approach provides a practical roadmap for spatially targeted, equitable interventions to safeguard urban habitability in a warming world.

## Methods

To assess the future temperature development in cities, we utilize downscaled climatological data from the CHELSA dataset^13^, which offers high-resolution (1 km) climate projections. We used MAT projections from the MPI-ESM^20,21^. The temperature projections are derived using bias-corrected global climate model outputs under several greenhouse gas concentration scenarios, specifically SSP1-2.6, SSP3-7.0, and SSP5-8.5. These scenarios correspond to varying levels of future emissions and mitigation efforts. The use of these scenarios allow us to project temperature changes at the urban scale, capturing local variations within specific cities. Additionally, to address uncertainties, we conducted supplementary analyses using SSP1-2.6 (low-emission scenario) and SSP3-7.0 (intermediate-emission scenario with high population growth).

For estimating future urban population exposure, we incorporated downscaled gridded population data based on the Shared Socioeconomic Pathways (SSPs) framework^10^. The SSP framework offers different pathways (SSP1-2.6, SSP3-7.0, SSP5-8.5) reflecting diverse socioeconomic development scenarios, each tied to varying assumptions about economic growth, urbanization, and population change. For our main analysis, we used population projections from SSP5-8.5^22^, which represents a pathway characterized by rapid growth and high urbanization rates. By downscaling these population datasets to align with our high-resolution climate projections^13^, we were able to quantify the number of people likely to be exposed to extreme temperatures in various urban areas under different future scenarios.

Furthermore, we incorporated remote sensing-derived morphological data on cities^1^, including information on urban form, density, and land use to delineate cities spatially throughout the world in a consistent and thus comparable way. With this consistent delineation of cities, we make the analysis independent of administrative demarcations, which have led to very heterogeneous spatial units through historical, political or other processes^1^. This helps to better understand how these factors interact with climate data and influence temperature developments.

To identify cities and populations falling outside the human climate niche, we used the threshold of $\hbox {MAT} =$ 29 °C as a benchmark^5^. This value has been established in prior research as a critical upper limit for human comfort and habitability. By analyzing the proportion of cities and their populations that are projected to exceed this temperature threshold under different future scenarios, we were able to quantify the potential impacts of rising temperatures on urban areas worldwide.

Finally, our analysis focused on three future time periods: 2011–2040, 2041–2070, and 2071–2100. We examined the spatial distribution of temperature changes for each period, as well as the projected population living in cities that will experience extreme temperatures. To calculate the populations for the time frame 2011–2040 we used the downscaled estimation^10^ for the year 2025, for the time frame 2041–2070 the estimated population for 2070 and for the time frame 2071–2100 the estimated population for 2100. These projections provide a comprehensive view of the potential risks cities face due to climate change and highlight areas where adaptation efforts will be most critical.

To further investigate the role of urban morphology in influencing extreme temperature outcomes, we conducted an analysis comparing different groups of LCZ shares between cities projected to exceed the critical threshold of 29 °C and those that do not. Therefore we used a global map^9^ of LCZ and clipped it using the morphological boundaries^1^ to calculate the share of each LCZ of the total respective urban area. For each group of LCZ classes, we separated the cities into two groups based on their projected mean temperature for the period 2071–2100: one group with values < 29 °C and another with values > 29 °C. This threshold was chosen following prior research^5^ which established 29 °C as a critical upper limit for human comfort and habitability.

Within each group of LCZ, we compared the distribution of LCZ shares across the two temperature groups using Welch’s t-test (which does not assume equal variances) to determine whether the differences in urban form were statistically significant. The analysis was performed both for the entire dataset and stratified by continent with cities with a MAT higher than 29 °C (Africa, Asia, North America, and South America). For visualization, we generated box plots for each LCZ class where, in a single subfigure, the box plots for the four continents and for the two temperature groups (< 29 °C vs. > 29 °C) are placed side by side. Significant differences (*p* < 0.05) between the temperature groups for a given continent and LCZ are highlighted with a star annotation above the corresponding pair of box plots.

This additional analysis enables us to assess whether specific urban forms (as indicated by the LCZ shares) are systematically associated with cities that are projected to experience extreme temperatures. The results provide further insight into the interplay between urban morphology and climate change impacts, informing targeted adaptation strategies that may help mitigate urban heat extremes.

To score adaptation strategy relevance, we imported an expert-curated LCZ-measure mapping matrix adapted from^11^ (Table 1) that assigns binary weights (0/1) for eight interventions—ranging from “Raise pervious-surface fraction” to “Control industrial heat emissions”—to each LCZ class.

**Table 1:** Measures per Local Climate Zone (LCZ) based on^11^.

Measure	LCZ	LCZ	LCZ	LCZ	LCZ	LCZ
	1	2	3	4	8	10
Optimize Urban Openness	1	1	1	0	0	0
Regulate Densification	1	1	1	0	0	0
Restrict Building Height	1	0	0	1	0	0
Raise Pervious Surface Fraction Ratio	1	1	1	0	1	0
Use Reflective Building Materials	1	1	1	1	1	1
Increase Green Space and Vegetation Cover	1	1	1	1	1	1
Increase Water Bodies	1	1	1	1	1	1
Reduce Environmental Pollution	1	0	0	0	1	1
Control Heat Emissions from Factories	1	0	0	0	1	1
Promote Sustainable Urban Forms	0	0	0	0	1	1

We aligned the mapping matrix to our LCZ columns, then computed per-city weighted scores via the dot product of LCZ shares $S_\text{LCZ}$ and mapping weights $w_\mathrm {LCZ-Measure}$:

1 $$\begin{aligned} Score = \sum _{\text{LCZ}}(S_\text{LCZ}\times w_\mathrm {LCZ-Measure}.) \end{aligned}$$

The resulting scores, appended to the city dataset, quantify each city’s potential responsiveness to each adaptation measure. We aggregated these scores by continent—summing for total potential and averaging for per-city potential.

To quantify each city’s aggregate economic capacity, we used gridded GDP (PPP) data at 30 arcsec resolution for 2020^12^. For each morphological city polygon^1^, we summed all grid-cell values within the polygon to obtain a total GDP PPP per city. These per-city GDP enable subsequent analyses of economic capacity versus projected MAT and adaptation-strategy scores.

Finally, during the preparation of this work the author used ChatGPT and DeepL to improve readability of some paragraphs. After using this tool, the authors reviewed and edited the content as needed and take full responsibility for the content of the published article.

## Supplementary Information

Supplementary Information 1.

Supplementary Information 2.

Supplementary Information 3.

Supplementary Information 4.

## Data Availability

All data sets used to perfom the study are publicly available. The shapefiles containing the spatial delineations of the morphological urban areas can be downloaded at https://doi.org/10.1016/j.rse.2019.111353. The downscaled climate models can be downloaded at https://chelsa-climate.org/downloads/. The downscaled population scenarios can be downloaded at https://figshare.com/articles/dataset/The_code_of_Projecting_1_km-grid_population_distributions_from_2020_to_2100_globally_under_shared_socioeconomic_pathways_/19609356/3?file=36928720. The gridded GDP maps can be downloaded at https://zenodo.org/records/13943886 The code to perform the analyses and to create the figures is available at https://github.com/johnfriesen/Urban-Climate-Scenarios.

## Code availability

The code to perform the analyses and to create the figures is available at https://github.com/johnfriesen/Urban-Climate-Scenarios.

## References

[1] Taubenböck, H. et al. A new ranking of the world’s largest cities-do administrative units obscure morphological realities?. *Remote Sens. Environ.* **232**, 111353 (2019).

[2] Intergovernmental Panel On Climate Change (Ipcc): Climate Change 2022 - Impacts, Adaptation and Vulnerability: Working Group II Contribution to the Sixth Assessment Report of the Intergovernmental Panel on Climate Change, 1st edn. Cambridge University Press. 10.1017/9781009325844 . https://www.cambridge.org/core/product/identifier/9781009325844/type/book Accessed 04 Nov 2024

[3] United Nations Human Settlements Programme (UN-Habitat): World Cities Report 2024 - Cites and Climate Action. https://unhabitat.org/sites/default/files/2024/11/wcr2024_-_full_report.pdf Accessed 04 Nov 2024

[4] Xu, C., Kohler, T. A., Lenton, T. M., Svenning, J.-C. & Scheffer, M. Future of the human climate niche. *Proc. Natl. Acad. Sci.* **117**(21), 11350–11355 (2020). 32366654 10.1073/pnas.1910114117 PMC7260949

[5] Lenton, T. M. et al. Quantifying the human cost of global warming. *Nat. Sustain.* **6**(10), 1237–1247 (2023).

[6] Klinger, B. A. & Ryan, S. J. Population distribution within the human climate niche. *PLOS Climate* **1**(11), 0000086 (2022).

[7] Taubenböck, H. et al. Global differences in urbanization dynamics from 1985 to 2015 and outlook considering IPCC climate scenarios. *Cities* **151**, 105117 (2024).

[8] Tuholske, C. et al. Global urban population exposure to extreme heat. *Proc. Natl. Acad. Sci.* **118**(41), 2024792118 (2021). 10.1073/pnas.2024792118 PMC8521713 34607944

[9] Demuzere, M. et al. A global map of local climate zones to support earth system modelling and urban scale environmental science. *Earth Syst. Sci. Data Discuss.* **2022**, 1–57 (2022).

[10] Wang, X., Meng, X. & Long, Y. Projecting 1 km-grid population distributions from 2020 to 2100 globally under shared socioeconomic pathways. *Sci. Data* **9**(1), 563 (2022). 36097271 10.1038/s41597-022-01675-x PMC9466344

[11] Rahmani, N. & Sharifi, A. Urban heat dynamics in local climate zones (lczs): A systematic review. *Build. Environ.* **267**, 112225 (2025).

[12] Kummu, M., Kosonen, M. & Masoumzadeh Sayyar, S. Downscaled gridded global dataset for gross domestic product (GDP) per capita PPP over 1990–2022. *Sci. Data* **12**(1), 178 (2025). 39885148 10.1038/s41597-025-04487-x PMC11782586

[13] Karger, D. N. et al. Climatologies at high resolution for the earth’s land surface areas. *Sci. Data* **4**(1), 1–20 (2017). 10.1038/sdata.2017.122 PMC5584396 28872642

[14] Vilanova, C., Ferran, J. S. & Concepción, E. D. Integrating landscape ecology in urban green infrastructure planning: A multi-scale approach for sustainable development. *Urban For. Urban Green.* **94**, 128248 (2024).

[15] Breuer, J. H., Friesen, J., Taubenböck, H., Wurm, M. & Pelz, P. F. The unseen population: Do we underestimate slum dwellers in cities of the global south?. *Habitat Int.* **148**, 103056 (2024).

[16] Celhay, P. & Undurraga, R. Location preferences and slums formation: Evidence from a panel of residence histories. *Reg. Sci. Urban Econ.* **97**, 103816 (2022).

[17] Selby, J., Hulme, M. & Cramer, W. There is no human climate niche. *One Earth* **7**(7), 1155–1157 (2024).

[18] Zhang, K. et al. Increased heat risk in wet climate induced by urban humid heat. *Nature* **617**(7962), 738–742 (2023). 37100919 10.1038/s41586-023-05911-1

[19] Karagiorgos, K. et al. Global population datasets overestimate flood exposure in Sweden. *Sci. Rep.* **14**(1), 20410 (2024). 39223219 10.1038/s41598-024-71330-5 PMC11368945

[20] Brun, P., Zimmermann, N. E., Hari, C., Pellissier, L. & Karger, D. N. Global climate-related predictors at kilometer resolution for the past and future. *Earth Syst. Sci. Data* **14**(12), 5573–5603 (2022).

[21] Gutjahr, O. et al. Max planck institute earth system model (mpi-esm1.2) for the high-resolution model intercomparison project (highresmip). *Geosci. Model Dev.lopment* **12**(7), 3241–3281 (2019).

[22] Schwalm, C. R., Glendon, S. & Duffy, P. B. Rcp8.5 tracks cumulative co2 emissions. *Proc. Natl. Acad. Sci.* **117**(33), 19656–19657 (2020). 32747549 10.1073/pnas.2007117117 PMC7443890

