# Supplementary material for: The world’s largest cities under climate change and their adaptive capacity to rising heat
Source: Sci Rep. 2025 Sep 23;15:32671. doi: 10.1038/s41598-025-19954-z (PMC12457604; doi:10.1038/s41598-025-19954-z)
Supplement: Supplementary file 4 — Supplementary Information 4. [file 41598_2025_19954_MOESM4_ESM.pdf]

Supplementary Material - The World's Largest Cities Under Climate Change and Their Adaptive Capacity to Rising Heat

John Friesen, Hannes Taubenböck

September 2025

Additional figures

SSP126

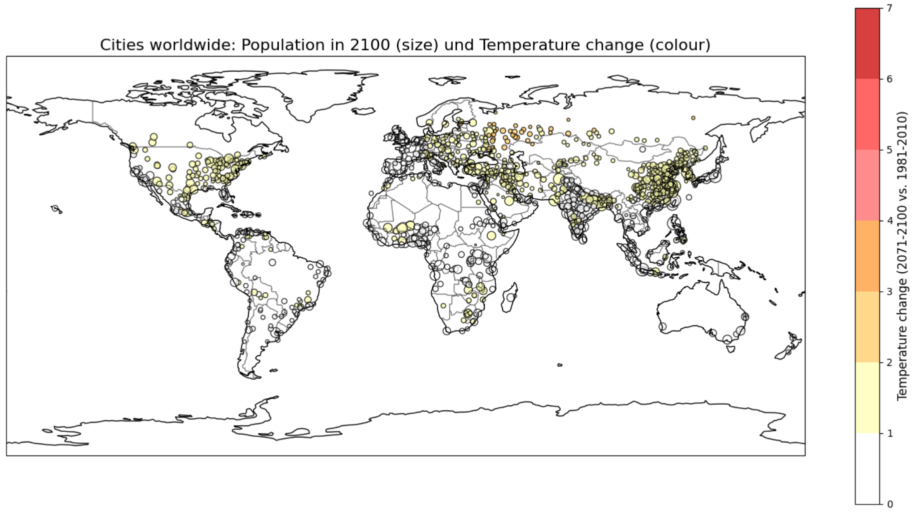

Figure S1: Changes in MAT in cities between reference time frame 1981-2010 and 2071-2100 according SSP126. The map was created with the code (Python 3.9 and the *cartopy* package) provided in the repository in the code availability statement in the paper.

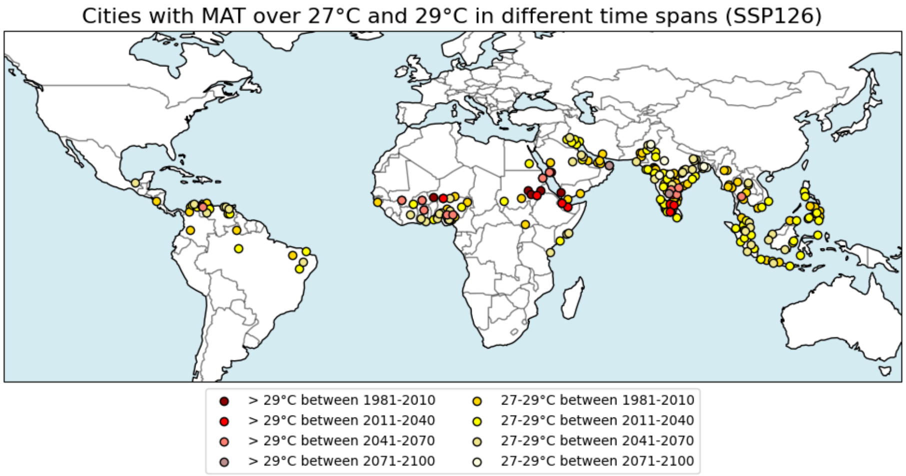

Figure S2: Geographical distribution of cities with MAT between 27 and 29 and over 29 degrees according to SSP126. The map was created with the code (Python 3.9 and the *cartopy* package) provided in the repository in the code availability statement in the paper.

SSP370

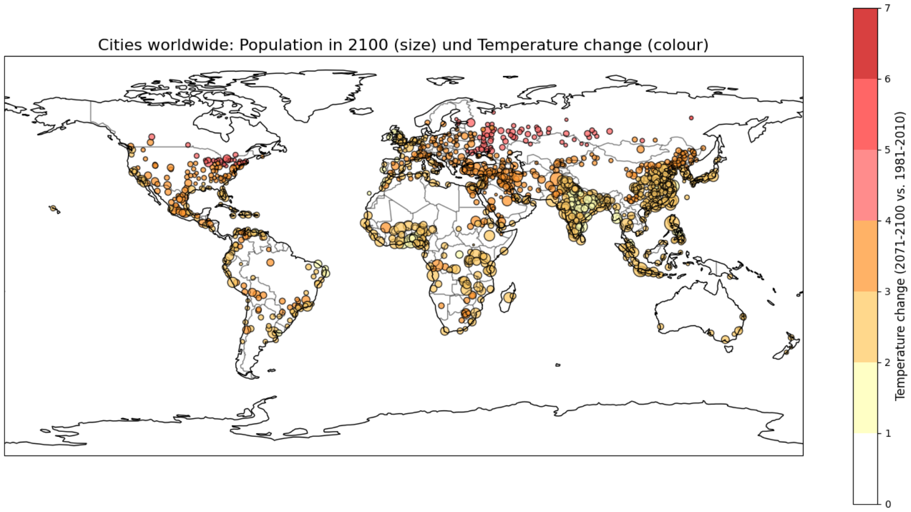

Figure S3: Changes in MAT in cities between reference time frame 1981-2010 and 2071-2100 according SSP370. The map was created with the code (Python 3.9 and the *cartopy* package) provided in the repository in the code availability statement in the paper.

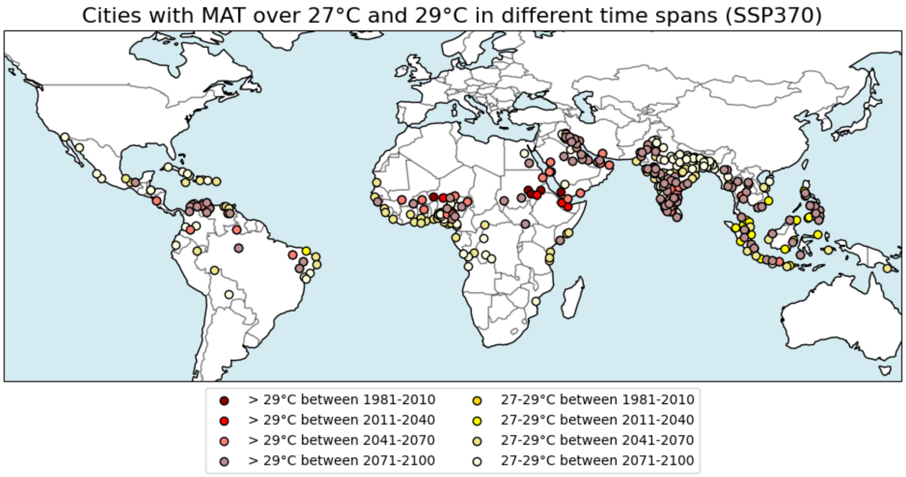

Figure S4: Geographical distribution of cities with MAT between 27 and 29 and over 29 degrees according to SSP370. The map was created with the code (Python 3.9 and the *cartopy* package) provided in the repository in the code availability statement in the paper.

SSP585

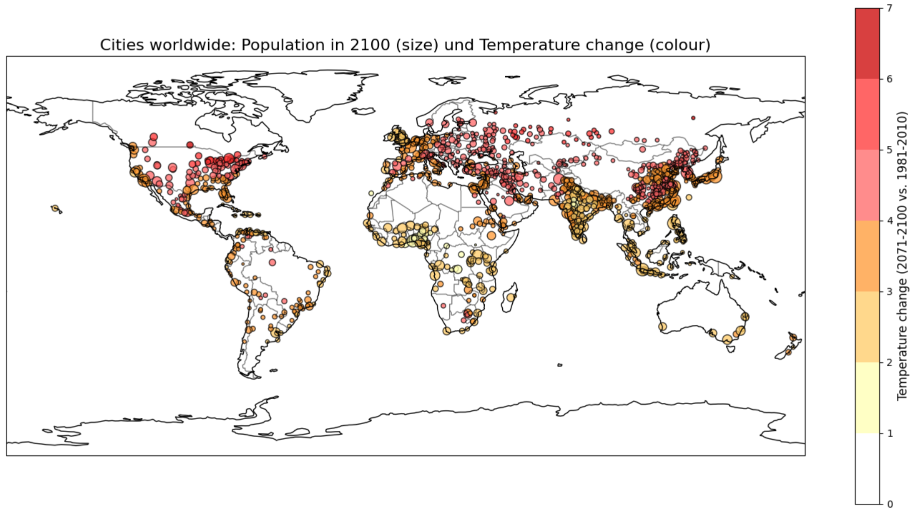

Figure S5: Changes in MAT in cities between reference time frame 1981-2010 and 2071-2100 according SSP585. The map was created with the code (Python 3.9 and the *cartopy* package) provided in the repository in the code availability statement in the paper.

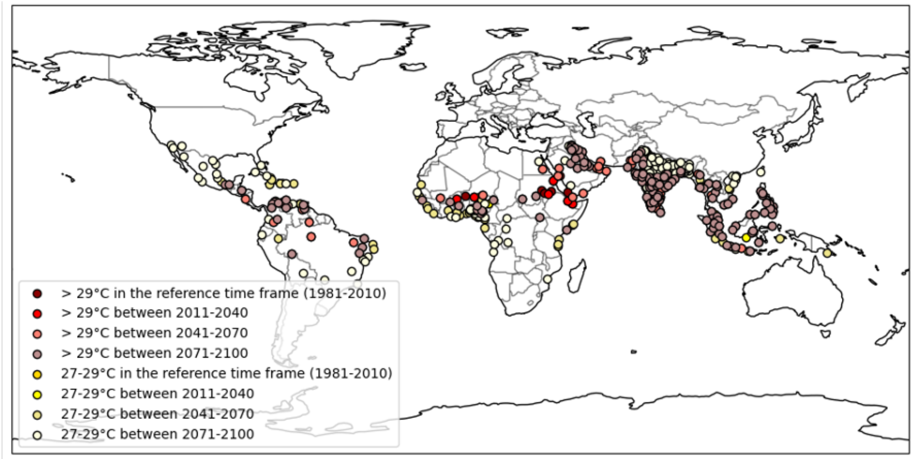

Figure S6: Geographical distribution of cities with MAT between 27 and 29 and over 29 degrees according to SSP585. The map was created with the code (Python 3.9 and the *cartopy* package) provided in the repository in the code availability statement in the paper.

Analysis Local climate zones

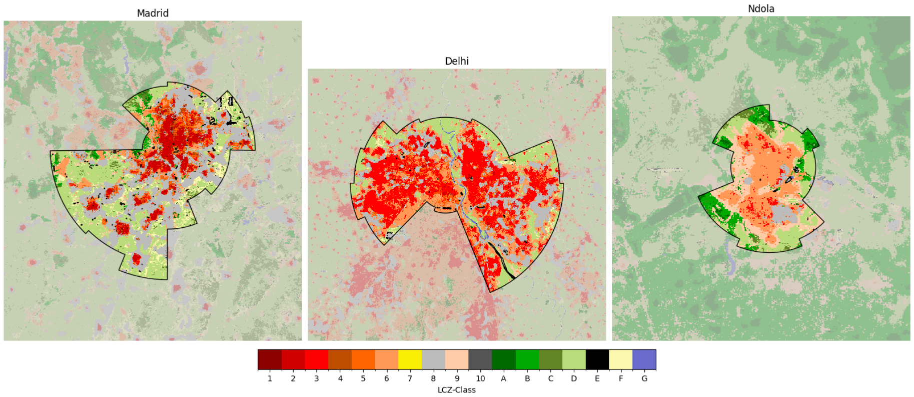

Figure S7: Geographical distribution of local climate zones in three exemplary cities with morphological urban areas. The map was created with the code provided in the repository in the code availability statement in the paper. The local climate zone data shown in the figure was taken from Google Earth Engine: ee.ImageCollection(RUB/RUBCLIM/LCZ/global lcz map/latest)

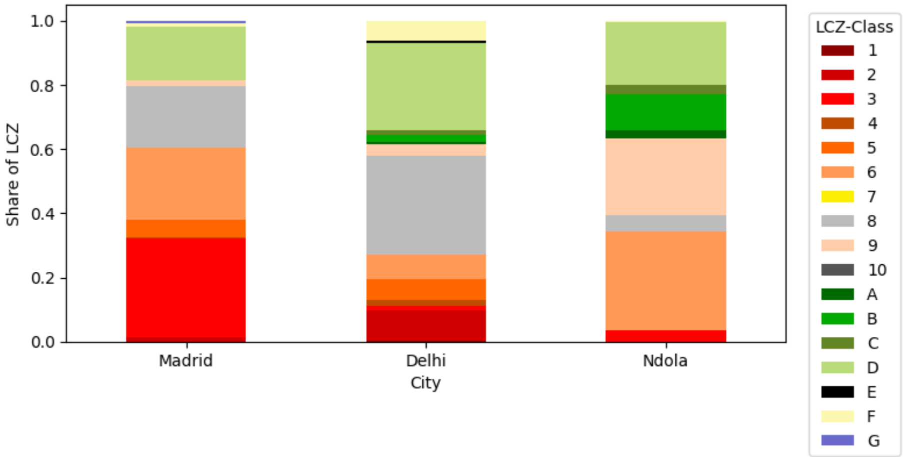

Figure S8: Share of local climate zones in three exemplary cities.
